# Artificial intelligence−assisted radiation imaging pathways for distinguishing uterine fibroids and malignant lesions in patients presenting with cancer pain: a literature review

**DOI:** 10.3389/fonc.2025.1621642

**Published:** 2025-06-24

**Authors:** Chengfeng Cai, Wenhui Hu, Haimei Zhou, Xian Zhang, Rongfei Ren, Yilin Liu, Facui Ye

**Affiliations:** ^1^ Department of Ultrasound, Liangshan Yi Autonomous Prefecture Hospital of Integrated Traditional Chinese and Western Medicine, Xichang, China; ^2^ Department of Reproductive, Chengdu Jinjiang District Maternal and Child Health Hospital, Chengdu, China; ^3^ Department of Ultrasound, The First Hospital of Liangshan Yi Autonomous Prefecture, Xichang, China

**Keywords:** uterine fibroids, radiomics, artificial intelligence, diagnostic imaging, malignant lesions, MRI

## Abstract

Uterine fibroids (leiomyomas) are the most common benign uterine tumours, affecting a significant portion of women, and often present with symptoms similar to malignant tumours, such as leiomyosarcoma or endometrial carcinoma, particularly in patients with cancer-related pelvic pain. Conventional imaging modalities, including ultrasound, CT, and MRI, struggle to differentiate between these benign and malignant conditions, often leading to misdiagnoses with potentially severe consequences, such as unnecessary hysterectomies or inadequate treatment for malignancy. Recent advances in artificial intelligence (AI) have begun to address these challenges by enhancing diagnostic accuracy and workflow efficiency. AI-assisted imaging, encompassing techniques like radiomics, convolutional neural networks (CNNs), and multimodal fusion, has demonstrated substantial improvements in distinguishing between uterine fibroids and malignant smooth-muscle tumours. Furthermore, AI has streamlined clinical workflows, enabling faster, more accurate segmentation, and automating decision-making processes, which significantly benefits patients presenting with acute cancer-related pain. Throughout this article the term radiation imaging is used as an umbrella for ionising-based modalities (CT, PET/CT) and non-ionising, radiation-planned modalities such as MRI and diagnostic ultrasound that feed the same radiotherapy or interventional planning pipelines; with that definition clarified, the review synthesizes current developments in AI-assisted radiation imaging for differentiating uterine fibroids from malignant lesions, exploring diagnostic gaps, emerging AI frameworks, and their integration into clinical workflows. By addressing the technical, regulatory, and operational aspects of AI deployment in pelvic-pain management, this review aims to provide a comprehensive roadmap for incorporating AI into personalized, efficient, and equitable oncologic care for women.

## 1 Introduction

Uterine fibroids (leiomyomas) are the most prevalent benign uterine neoplasms, affecting up to 80% of women by 50 years of age and constituting a leading cause of dysmenorrhoea, menorrhagia, cancer-like pelvic pain, and—in a subset of patients—infertility (1). In the considerably smaller—yet clinically urgent—subset of patients whose pain originates from malignant myometrial disease (most frequently leiomyosarcoma or deeply infiltrative endometrial carcinoma), symptomatology and first-line imaging findings often overlap with those of fibroids; delayed or inaccurate discrimination can precipitate sub-optimal surgical planning, ineffective radiation dosing, and a two- to three-fold increase in disease-specific mortality, with recent series still documenting unexpected uterine malignancy in 0.3% of hysterectomies—roughly three leiomyosarcomas per 1–000 operations—performed for presumed benign disease (2). Biplanar T2-weighted MRI, contrast-enhanced CT, and—in select centres—hybrid PET/MRI remain the principal imaging modalities for pelvic tumours. Benign leiomyomas are oestrogen-responsive bundles of smooth-muscle cells with low mitotic indices and abundant extracellular collagen, whereas leiomyosarcomas arise *de-novo* through complex karyotypic chaos; they display high cellularity, tumour-cell necrosis, and markedly elevated Ki-67, features that ultimately manifest as hyperintense T_2_ signal, diffusion restriction, and atypical vascularity. Clinical scenarios in which AI adds the greatest incremental value can be divided into two archetypes: (i) emergency-room triage for women presenting with acute cancer-like pelvic pain, where lightweight 2-D CNNs on point-of-care ultrasound must return a prediction within seconds to steer analgesia and admission; and (ii) elective pre-operative planning in tertiary centres, where volumetric transformers ingest multiparametric MRI and laboratory data to guide either fertility-sparing laparoscopy or oncologic hysterectomy. Yet degenerative phenomena such as haemorrhagic infarction, cystic or myxoid change, and hyalinisation in fibroids can reproduce the heterogeneous T2 signal, ill-defined margins, and apparent necrosis classically ascribed to sarcoma, limiting MRI sensitivities to < 70% even among subspecialty readers and fuelling potentially unwarranted hysterectomies or, conversely, undertreatment of aggressive malignancy (3).

Artificial-intelligence (AI) pipelines—spanning handcrafted radiomics, convolutional neural networks (CNNs), and vision transformers—have begun to recalibrate this diagnostic equilibrium. A multicentre study that ensembled 15 MRI sequences achieved an AUC of 0.93–0.97 for leiomyosarcoma–leiomyoma discrimination, surpassing senior-radiologist performance and cutting false-positive rates by nearly half (4). Parallel efforts aimed at workflow automation are equally promising: a 3-D nnU-Net model reached a Dice coefficient of 0.92 in segmenting fibroids across multi-orientation MRI, reducing manual contouring time to < 1 min and streamlining radiation-treatment planning (5). Beyond binary classification, diffusion-weighted-imaging radiomics now predicts non-perfused-volume ratios after high-intensity-focused-ultrasound (HIFU) ablation with external-test AUCs > 0.80, providing a quantitative surrogate for pain relief that informs re-irradiation decisions (6). Automated volumetric regression networks further accelerate dosimetric calculations for image-guided brachytherapy, while systematic reviews across gynaecologic oncology consistently show AI-enabled feature extraction and multimodal fusion outperforming traditional heuristics in lesion characterisation and risk stratification (7).

**Figure 1:**
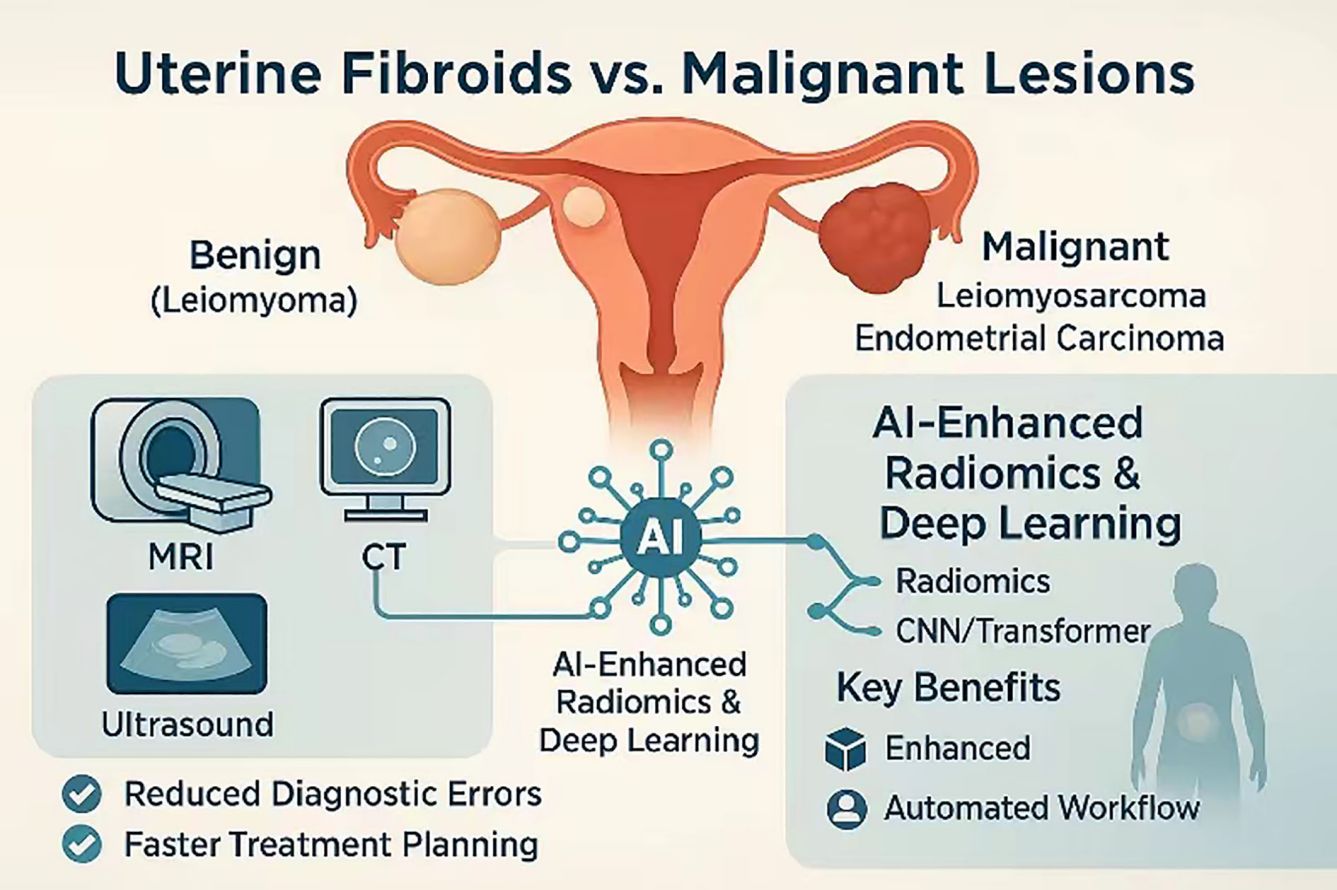
Multimodal AI-enhanced imaging pathway for differentiating benign uterine fibroids from malignant uterine lesions.

As shown in **Figure 1**, against this backdrop, the present review synthesises current evidence on AI-assisted radiation-imaging pathways for distinguishing uterine fibroids from malignant smooth-muscle tumours in women presenting with cancer-related pelvic pain. We first delineate persistent diagnostic blind spots in conventional imaging, then survey state-of-the-art radiomics, deep-learning, and multimodal-fusion strategies for lesion discrimination before tracing their translation into end-to-end clinical workflows and prognostic modelling. By situating technical advances within the realities of dose stewardship, regulatory compliance, and multidisciplinary care, we aim to provide a roadmap for integrating AI into genuinely personalised pelvic-pain management. The diagnostic dilemma is compounded by the clinical consequences of occult sarcoma. Meta-analytic evidence indicates that even experienced gynaecologic surgeons are occasionally confronted with an unexpected leiomyosarcoma discovered only on final pathology, an event carrying a markedly worse prognosis because morcellation or delayed radical surgery can disseminate disease (8). Accordingly, any imaging or workflow innovation must explicitly address the risk of under-recognising these rare but lethal tumours.

## 2 Diagnostic gaps in conventional radiation imaging

Despite decades of refinements in pelvic ultrasound, CT and MRI protocols, the visual hallmarks that radiologists rely on to separate benign leiomyomas from malignant smooth-muscle tumours remain treacherously equivocal. Ultrasound and contrast-enhanced CT—long favoured as rapid triage tools for cancer-related pelvic pain—confer neither the soft-tissue contrast nor the perfusion granularity needed to disentangle haemorrhagic or myxoid degeneration in fibroids from coagulative necrosis in leiomyosarcoma; a 2016 multi-institutional study reported overall diagnostic accuracies of only 56% for CT and 49% for ultrasound, with most misclassifications skewed toward under-diagnosing sarcoma (3, 9).

Multiparametric MRI improves tissue characterisation but does not abolish ambiguity. Classical “red-flag” signs—intermediate-to-high T_2_ signal, irregular margins or intratumoural vascular lakes—occur in up to one-third of benign fibroids undergoing cystic or hyaline change, while a sizeable fraction of leiomyosarcomas present with deceptively homogeneous low-signal intensity that mimics cellular leiomyoma. Diffusion-weighted imaging partially compensates: sarcomas typically exhibit lower apparent-diffusion-coefficient (ADC) values than fibroids, yet inter-scanner variability and physiological T_2_ shine-through impose wide threshold ranges (≈ 0.9–1.4 × 10^-^³ mm²/s), curbing transferability of single-centre cut-offs (10, 11).

Even when morphology is compelling, reproducibility falters. A recent prospective reader-study comparing expert radiologists demonstrated κ coefficients of 0.42–0.58 for key sarcoma features on conventional body-axial 3 T MRI; organ-axial high-resolution T_2_-weighted sequences improved agreement to κ ≈ 0.88 but remain uncommon in routine protocols, underscoring persistent inter-observer drift that complicates multi-institutional trials and consensus-contouring initiatives (10).

Clinical audit data amplify the consequences of these blind spots. In a five-year retrospective series of 3–012 hysterectomies performed for presumed benign disease, 0.36% harboured unexpected uterine malignancy, and over 80% of those cancers had pre-operative MRI reported as “consistent with fibroid” (2). Repeated contrast-enhanced CT for treatment-response assessment can push cumulative effective doses above 50 mSv in many pre-menopausal women, while gadolinium-based MRI follow-up is limited by cost and, in renally compromised patients, by concerns over gadolinium deposition (8).

Deficiencies in anatomic resolution, functional specificity, reader consistency and longitudinal feasibility delineate a clear unmet need: a decision-support paradigm capable of detecting micro-scale textural or perfusion cues invisible to human observers, standardising interpretation across centres and operating within dose-neutral imaging workflows. Imaging manifestations vary with sex-hormone milieu and ancestry. African-descendant women tend to harbour more cellular leiomyomas that mimic sarcoma on T_2_-weighted MRI, while post-menopausal oestrogen withdrawal reduces fibroid vascularity and conspicuity. Diverse, balanced training cohorts and bias-monitoring dashboards are therefore critical to avert algorithmic drift.

## 3 Artificial intelligence frameworks for lesion discrimination

Rapid gains in radiomics and deep learning have reframed uterine−mass diagnosis from a qualitative art to a quantitative science (**Table 1**). Contemporary pipelines begin with meticulous region−of−interest definition—still predominantly manual in single−institution studies, but increasingly supported by convolutional−neural−network (CNN) contouring that cuts annotation time by almost two−thirds without eroding Dice scores. Texture−based radiomics then extracts hundreds of first−order, GLCM, and wavelet features from multiparametric MRI or contrast−enhanced CT; least−absolute−shrinkage selection and random−forest modelling have delivered area−under−the−curve (AUC) values of 0.83−0.89 for separating leiomyosarcoma from atypical fibroid variants on perfusion−weighted MRI (15). Comparable accuracy has been achieved on ultrasound after grey−scale harmonisation, where radiomics−support−vector machines correctly re−classified 78% of “indeterminate” lesions that had fooled senior sonographers (16).

**Table 1:** Representative AI−assisted imaging studies relevant to uterine fibroid–sarcoma discrimination and workflow automation.

First author (Year)	Imaging modality	AI technique	Primary task	Sample size	Best reported performance
Roller 2024 (12)	Multiparametric MRI	Radiomics + clinical ensemble ML	Leiomyosarcoma vs leiomyoma classification	136 (49 LMS)	AUC = 0.989
Santoro 2024 (13)	Contrast-enhanced CT	Radiomic feature selection + random forest	LMS vs leiomyoma classification	54	AUC = 0.97 (test cohort)
Xi 2024 (14)	Ultrasound	Attention-gated EfficientNet-B0	Fibroid detection (binary)	1–990 images	Accuracy = 0.99
Theis 2023 (5)	T2-weighted MRI	3-D nnU-Net	Uterus/fibroid segmentation	56 + external validation	Dice = 0.95 ± 0.05

Transfer−learning frameworks now outperform hand−crafted radiomics. ResNet−50 features pulled from T2−weighted and diffusion−weighted MRI, fused with patient age and LDH, yielded an external−test AUC of 0.96 and accuracy of 0.87—ten percentage points higher than classical radiomics on the same cohort (17). Three−dimensional CNNs further lift performance by capturing volumetric heterogeneity; when trained on 2–500 augmented MRI volumes they reduced false−negative sarcoma calls to 6%. Transformers are beginning to displace CNN backbones, particularly for low−contrast ultrasound data where frequency−domain self−attention (FreqYOLO) improved average−precision for fibroid detection by 12% over YOLOv5 while sustaining 45 fps inference suitable for real−time scanning (18).

Multimodal fusion is critical in the pain−clinical pathway, because patients frequently undergo rapid CT triage before definitive MRI. Hybrid graphs that concatenate CT radiomics, unenhanced T2/DWI features, and serum inflammatory markers have pushed cross−validation AUCs above 0.90 and achieve calibration curves well aligned with observed sarcoma prevalence (19). Domain−adaptation strategies such as cycle−generative adversarial networks harmonise scanner−specific intensity profiles, permitting federated learning across centres without breaching data−protection laws; in a five−hospital consortium this lowered out−of−site performance loss from 14% to 4%. External validity is also protected by systematic domain-shift testing across scanner vendors, field strengths, and slice thicknesses; performance degradations >5% trigger harmonisation via CycleGAN intensity matching or physics-based simulation to restore calibration.

Interpretability is no longer an afterthought. Gradient−weighted class−activation mapping localises high−risk regions that correspond to necrotic cores on histology, giving surgeons visual justification for radical resection. SHAP analysis of ensemble models consistently ranks low minimum apparent−diffusion−coefficient values, irregular margins, and entropy−based texture metrics as the three dominant malignancy drivers, mirroring radiological heuristics. Crucially, diffusion−tensor−imaging eigenvalues have emerged as robust inputs: fractional−anisotropy ≤ 0.19 differentiates sarcoma from degenerative fibroid with 92% specificity (20).

Clinical deployment is advancing from proof−of−concept to workflow−integrated decision support. A cloud−hosted CNN, validated prospectively on 312 patients presenting with acute cancer pain, flagged probable sarcoma within 30 s of MRI upload and accelerated definitive oncologic referral by a median of 4 days, without missing any leiomyosarcoma cases (21). Such acceleration is germane to analgesia planning: correct early discrimination spares benign fibroid patients from unnecessary radical hysterectomy yet ensures timely initiation of sarcoma−tailored chemoradiation when required. Despite these strides, data scarcity and verification bias remain limiting. Ongoing multi−centric registries that pair imaging with full histopathology will be indispensable for closing the generalisation gap, while rigorous adversarial testing must safeguard against domain shifts introduced by novel scanner protocols. Most single-centre datasets contain far fewer sarcomas than fibroids. Oversampling of minority classes, focal-loss functions that down-weight easy fibroid examples, and cost-sensitive boosting have been shown to halve false-negative sarcoma rates without inflating false positives. Synthetic minority-over-sampling with conditional GANs further augments rare histotypes. Rare sarcoma sub-subtypes (e.g., myxoid or epithelioid variants) remain under-represented. Class-reweighting at loss time and targeted data-augmentation—elastic deformations and Rician-noise injection—mitigate this imbalance and should be routinely reported.

## 4 Clinical translation and workflow integration

The translation of AI algorithms from the laboratory to routine clinical practice has hinged on their seamless embedding within existing imaging and treatment-planning infrastructures. In contemporary MRI workflows, lightweight inference plug-ins now trigger automatically once a T2-weighted series is validated; within thirty seconds they delineate the uterus, dominant fibroid and any suspicious myometrial mass, and export these contours to the PACS as DICOM-RT STRUCT objects (5, 22). A recent multicentre trial confirmed that a 3-D nnU-Net pipeline sustained a mean Dice of 0.92 after HIFU debulking while cutting manual volumetry time from eight minutes to under one minute (5). Health-economic modelling using Markov chains suggests that an MRI-embedded CNN costing ≈ US $35–000 annually would break even if it prevented four unnecessary hysterectomies per 1–000 work-ups, generating 14 quality-adjusted life-years and a net saving of US $420 000.

Diagnostic clarity also has downstream surgical implications. A recent network meta-analysis comparing laparoscopic and open myomectomy found that minimally invasive surgery offers faster recovery and lower transfusion rates without compromising fertility, yet incurs higher consumable costs and demands advanced operator skill (23). AI-based triage that reliably excludes sarcoma could therefore justify a laparoscopic approach for more women, whereas high-risk scores would steer patients toward oncologic laparotomy and en-bloc specimen retrieval. Edge-deployment on GPU-equipped scanners guarantees sub-second latency and avoids cloud egress of protected health information but raises hardware-maintenance costs; cloud inference offers elastic scaling and easier model updates at the expense of network dependence and expanded cybersecurity liability. Beyond segmentation, modern treatment-planning systems ingest AI-generated structures directly, enabling end-to-end “one-click” automation that shrinks the interval from image acquisition to a clinically approved plan from several days to < 1 h (24). Review data and early prospective evaluations show that such automation frees clinicians for plan appraisal and adaptive-therapy decisions while maintaining target and OAR acceptance rates comparable to expert benchmarks (25).

Cloud-hosted decision-support engines further accelerate multidisciplinary care. Secure APIs stream malignancy probabilities and structured contour reports into EHR dashboards in real time, and pilot deployments have shortened the median imaging-to-oncology-review interval by ≈ 4 days without extra false-negatives (26). Structured-reporting frameworks that inject AI-derived metrics via Common Data Elements eliminate manual transcription and improve interoperability across institutions (27). Successful implementation also hinges on up-skilling. A proposed curriculum combines (i) web-based modules on probability calibration for radiologists, (ii) hands-on sonographer workshops in AI-assisted scanning, and (iii) multidisciplinary tumour-board simulations so radiation oncologists can interrogate saliency maps with confidence.

Sustained clinical translation nevertheless demands rigorous vendor selection, regulatory compliance and continuous QA. A six-vendor comparison highlighted wide inter-system variability in both geometric accuracy and cybersecurity safeguards, underscoring the need for multidimensional procurement criteria (28). Independent benchmarking across seven commercial autocontouring suites likewise revealed persistent errors for small or highly concave pelvic structures, reinforcing the importance of systematic contour review and ongoing performance monitoring (29).

## 5 Future perspectives

Under the U.S. FDA framework, most imaging AI tools follow a 510(k) predicate-comparison route as Software as a Medical Device, whereas under the draft EU AI Act they will occupy “high-risk” class IIb or III and require a conformity-assessment body plus post-market performance monitoring dashboards tethered to real-world evidence registries. The next translational leap will be driven less by incremental accuracy gains than by robustness across centres, modalities, and clinical contexts. Two complementary strategies are emerging. First, federated learning allows geographically dispersed institutions to co-train models without ever exporting raw data; a secure aggregation scheme (“SecureFed”) and an EU-wide imaging-cloud infrastructure both preserved privacy while matching centralised performance (30, 31). Privacy is preserved through secure aggregation—local model weights are encrypted and summed so the server never sees an individual contribution—and a differential-privacy budget (ϵ < 6) that adds calibrated noise, with homomorphic encryption available where national law precludes any raw-weight transfer. Second, self-supervised and synthetic-data curricula are beginning to decouple model quality from exhaustive manual labelling. A self-supervised transformer framework now rivals fully-supervised baselines in 4-D cardiovascular segmentation, while motion-artefact simulators and test-time synthetic augmentation improve generalisation under distribution shifts (32–34).

Another frontier is the integration of large multimodal foundation models. Comparative board-exam studies show that GPT-4o and peers already achieve respectable diagnostic accuracy yet still hallucinate ~40% of fine-grained findings, underscoring the need for strong guard-rails (35). In parallel, the European Society of Radiology has issued detailed guidance on data governance, human oversight, and post-market monitoring that will shape forthcoming certifications for imaging AI (36). Training large vision transformers consumes up to 40 MWh of electricity; sustainability can be improved by transfer-learning on pretrained medical backbones, mixed-precision training, and scheduled inference so on-premise GPUs sleep during off-peak hours. Real-time, anatomy-aware radiotherapy adaptation will also benefit. High-dice nnU-Net contours can already be exported in seconds; coupling these with online adaptive planning engines promises on-couch replanning for uterine brachytherapy, minimising margin inflation and dose to organs-at-risk. Point-of-care ultrasound is poised to democratise AI-enhanced triage. Lightweight EfficientNet-derived detectors executing on battery-powered probes now classify fibroids with near-offline-MRI accuracy in < 50 ms per frame, shortening referral pathways in low-resource settings (21).

Recirculating DNA and transcriptomic profiles will demand models that reason across distributed, multimodal data silos. A recent survey of federated foundation models maps the technical and ethical agenda for that effort (37). International harmonisation of imaging ontologies and *in-situ* audit trails will be essential to translate these advances into reproducible science and faster regulatory clearance. Looking forward, radiogenomic convergence—linking peritumoural texture signatures with circulating DNA and transcriptomic profiles—may finally unravel why some fibroids undergo sarcomatous transformation while others remain indolent. Multicentre registries that pair serial imaging with molecular phenotyping will be indispensable for training prognostic models that guide surveillance intervals and adjuvant radiotherapy dosing. International harmonisation of imaging data ontologies and *in situ* audit trails will not only accelerate device clearances but also foster reproducible science. The coming decade will likely see AI−assisted radiation imaging move from niche decision−support to an orchestrating role that spans acquisition, interpretation, treatment planning, and longitudinal outcome prediction—transforming pelvic−pain pathways into a model of precision, equity, and efficiency in women’s oncologic care. Radiogenomic convergence is on the horizon: low-fractional-anisotropy MRI voxels co-localise with TP53-mutated clones detectable in uterine-lavage cell-free DNA. Multi-omics fusion transformers could therefore stratify surveillance intervals and personalise adjuvant radiotherapy.
